# Congenital Variants of Gastrointestinal Rotation Found at Resection of Hepatopancreatobiliary Tumors: A Case Series with Review of the Literature

**DOI:** 10.1089/crpc.2015.29010.dwr

**Published:** 2016-01-01

**Authors:** David W. Rittenhouse, Michael J. Pucci, Jennifer L. Brumbaugh, Charles J. Yeo, Harish Lavu

**Affiliations:** Department of Surgery, Jefferson Pancreas, Biliary and Related Cancer Center, Thomas Jefferson University, Philadelphia, Pennsylvania.

**Keywords:** cholangiocarcinoma, gastrointestinal malrotation, pancreatic ductal adenocarcinoma

## Abstract

**Background:** Gastrointestinal malrotation arises from intrauterine events that occur early in the first trimester of gestation, and can result in a midgut volvulus that classically presents in the neonatal period with bilious emesis. Gastrointestinal malrotation can present clinically with symptoms such as chronic abdominal pain or bowel obstruction, or remain completely asymptomatic only to be discovered as an incidental finding much later in life during surgical exploration for other diseases. We sought to identify the prevalence of gastrointestinal malrotation in patients undergoing surgical exploration for hepatopancreatobiliary (HPB) malignancy and describe the operative considerations of these cases.

**Case Presentation:** We performed a retrospective review of our prospectively acquired HPB surgery database from January 1, 2006, to December 1, 2013. We identified three cases of gastrointestinal malrotation out of a total of 1220 HPB cases reviewed, which represents 0.2%. We found two cases of gastrointestinal malrotation in the setting of pancreatic ductal adenocarcinoma and one case in the setting of cholangiocarcinoma. All three patients underwent exploratory laparotomy with resection of their respective primary tumors. We searched the English literature for cases of HPB malignancy in the setting of gastrointestinal malrotation.

**Conclusion:** Our case series and review of the literature underscore the rarity and complexity of these cases.

## Introduction

Abnormalities in gastrointestinal rotation occur fairly commonly in the general population with an incidence of 1/200 to 1/500 live births. They arise from events that occur *in utero* that interrupt normal gastrointestinal rotation and subsequent fixation to the retroperitoneum, which normally occurs during the 4th to 10th weeks of fetal development. Up to 55% of individuals with gastrointestinal malrotation present within the first week of life and up to 80% present within the first month of life.^1^

Symptoms of gastrointestinal malrotation in the neonate typically include either bilious emesis or other clinical and radiographic evidence of high-grade small-bowel obstruction.^2^ Concurrent congenital anomalies have been reported in up to 42% of patients with gastrointestinal malrotation, with the most common being duodenal webs/atresia (11%), Meckel's diverticulum (11%), and omphalocele (5%).^3^

Although most cases of intestinal malrotation are discovered early, abnormalities of gastrointestinal rotation can persist into adulthood before becoming symptomatic. They may also be encountered incidentally at the time of workup for other diseases. Gastrointestinal malrotation in the adult can either present acutely with abdominal pain and emesis secondary to intestinal volvulus or it can present more insidiously with chronic vague abdominal pain and nausea typically from partial obstruction from intraperitoneal adhesions or “bands.”

Whether gastrointestinal malrotation in the adult predisposes patients to intestinal, pancreatic, or biliary tract malignancy is unclear. Campbell et al. reviewed all of the cases of gastrointestinal malrotation at one institution and found a high association of biliary tract anomalies in addition to one case of pancreatic cancer.^1^ In this study, we report three cases of gastrointestinal malrotation that were discovered during operation for hepatopancreatobiliary (HPB) malignancy. Two of the patients had a concomitant pancreatic ductal adenocarcinoma (PDA) and one patient had a cholangiocarcinoma. We detail the clinical course of these patients, provide a discussion of the embryology that leads to such anomalies, and put this series in context with the English literature.

## Methods

We queried retrospectively our prospectively maintained HPB surgery database at the Thomas Jefferson University Hospital for cases of HPB malignancy with concurrent gastrointestinal malrotation from January 1, 2006 to December 1, 2013. We analyzed patient demographics, preoperative studies, operative variables, postoperative complications, postoperative hospital length of stay, adjuvant therapy, and survival. For the literature review, we searched the English literature for all cases of HPB malignancy with concurrent abnormalities of gastrointestinal rotation from 1957 to 2013. We performed a PubMed search of the terms “pancreatic ductal adenocarcinoma,” “cholangiocarcinoma,” and “gastrointestinal malrotation.”

## Results

We identified three instances of patients who underwent surgical resection of HPB malignancy with concurrent gastrointestinal malrotation during the study period at our institution. Their cases are presented hereunder.

### Case 1

**FIG. 1.**
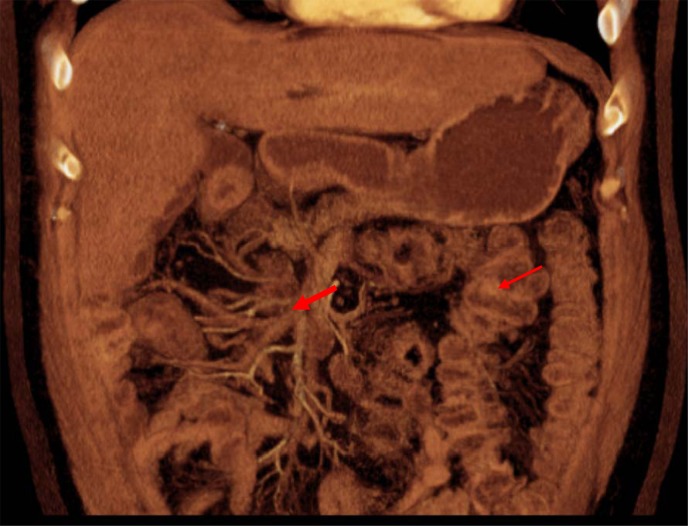
A magnetic resonance imaging/magnetic resonance cholangiopancreatography showing the third portion of the duodenum not crossing to the left of the SMA and SMV. The entire colon was to the left of the SMA and SMV. Thick arrow, third portion of duodenum; thin arrow, colon. SMA, superior mesenteric artery; SMV, superior mesenteric vein.

A 63-year-old man presented to the emergency department with a 1-month history of intermittent nausea, chills, and jaundice. Preoperative laboratories revealed an elevated total bilirubin and alkaline phosphatase. Serum tumor marker analysis revealed a normal carcinoembryonic antigen (CEA) but an elevated CA 19-9. The patient underwent a magnetic resonance imaging (MRI)/magnetic resonance cholangiopancreatography that revealed thickening of the proximal common bile duct with proximal biliary dilatation and an absence of distant disease. Incidentally noted on the MRI was that the third portion of the duodenum did not cross to the left of the superior mesenteric artery (SMA) and superior mesenteric vein (SMV), and the majority of the small intestine lies in the patient's right abdomen, whereas the large intestine lies in the left. Also, the SMA lies to the right of the SMV consistent with gastrointestinal malrotation (Fig. 1). The patient underwent endoscopic retrograde cholangiography with biliary endoprosthesis placement for decompression. Upon surgical exploration, a mass was identified in the proximal common bile duct (with no evidence of disseminated disease) and gastrointestinal malrotation was confirmed. Oncological resection of the extrahepatic biliary tree was performed from the level of the bifurcation of the right and left hepatic ducts to the intrapancreatic portion of the bile duct, along with a portal lymphadenectomy. A biliary enteric reconstruction was created with a Roux-en-Y hepaticojejunostomy. To compensate for the malrotation, the proximal jejunum was divided 50 cm distal to where the duodenum exited the retroperitoneum and the 50 cm Roux limb was brought up in a right paracolic position rather easily, as the hepatic flexure of the colon was absent, such that it simply rested over the top of the duodenum. An end-to-side hepaticojejunostomy was formed in a single layer, and the Roux limb was tacked down to the retroperitoneum to prevent intestinal herniation. Specimen pathology revealed a poorly differentiated adenocarcinoma of the common bile duct with negative surgical margins with negative nodal disease. The patient's postoperative course was complicated by a prolonged ileus, and he was discharged to home on postoperative day 16. The patient underwent 5-fluorouracil-based adjuvant chemotherapy and radiation therapy. He unfortunately developed recurrent malignant disease and died 23 months postresection.

### Case 2

**FIG. 2.**
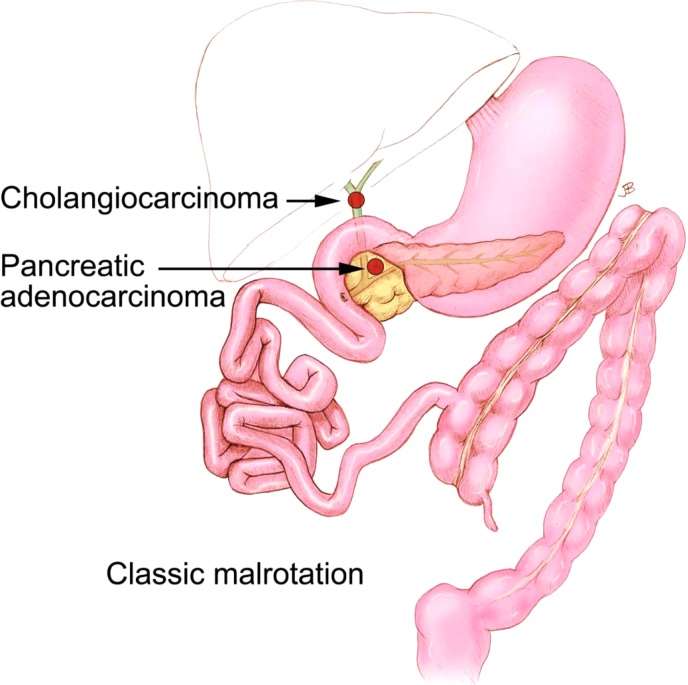
An illustration showing a classic gastrointestinal malrotation, with the entire colon on the left side of the abdomen. The location of malignant involvement from Cases 1 (cholangiocarcinoma) and 2 (pancreatic adenocarcinoma) is delineated.

A 47-year-old man presented to his primary care physician with a 3-month history of intermittent epigastric abdominal pain, nausea, and vomiting. Routine laboratory analysis revealed normal liver function. The patient underwent an abdominal computed tomography (CT) scan, which revealed a 4.2 × 4.1 × 4 cm mass in the head and uncinate process of the pancreas. The scan also showed that the patient's entire colon was on the left side of the abdomen and the small bowel to the right (Fig. 2). Tumor marker analysis showed a normal CEA level and an elevated CA 19-9 level. At operative exploration, the fourth portion of the duodenum was in its normal position; however, the proximal jejunum immediately traveled to the patient's right side and most of the small bowel was to the right of the ascending colon and the cecum located in the pelvis. The SMA was rotated closer to the uncinate process and was encased by tumor making the lesion unresectable. A palliative antiperistaltic gastrojejunostomy and Roux-en-Y hepaticojejunostomy bypass were performed. The jejunal limb was not brought up in the usual retrocolic manner but in a right paracolic position due to the abnormal colonic location. Intraoperative biopsy of the palpable mass revealed a moderately differentiated PDA. The patient underwent gemcitabine-based chemotherapy and radiation postoperatively with successful downstaging of the tumor. He underwent operative re-exploration with successful resection of his tumor through a classic pancreaticoduodenectomy ∼12 months after his initial exploration. Pathology of the resected specimen revealed a PDA with perineural tumor involvement at the uncinate process margin. Three out of the 13 lymph nodes examined were positive for metastatic disease. The patient was discharged from the hospital on postoperative day 8. He developed recurrent malignant disease and died 10 months postresection, that is 22 months following the initial exploration.

### Case 3

**FIG. 3.**
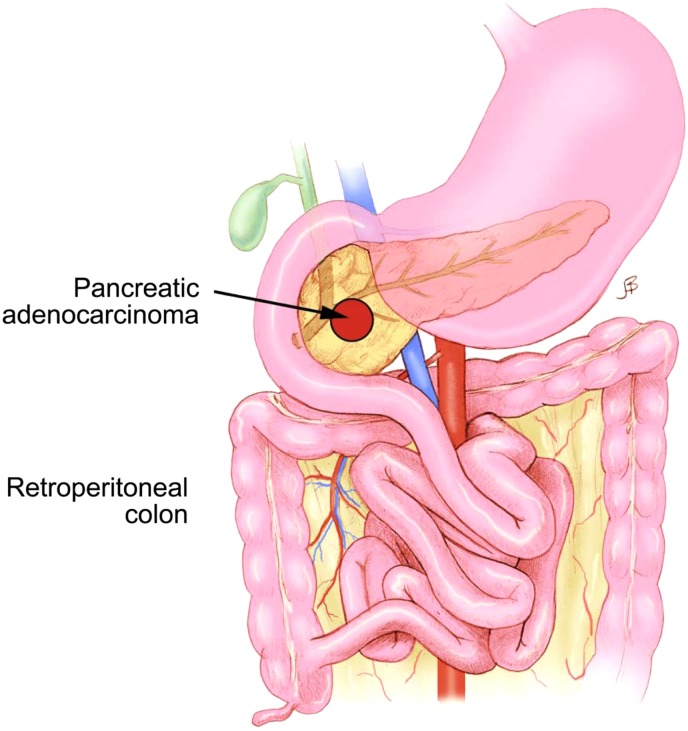
An illustration showing a reversed rotation of the gastrointestinal tract. The SMA lies ventral to the transverse colon. A pancreatic head mass is seen.

A 67-year-old Caucasian woman presented to her primary care physician with a 1-year history of epigastric abdominal pain, occasional nausea, and emesis, and an unintentional 10 pound weight loss. An abdominal CT scan showed a 4.5 × 4.1 cm periampullary mass arising from the pancreas without evidence of metastatic disease. Interestingly, the CT scan also showed the colon to be in a dorsal position in relation to the SMA (Fig. 3). Laboratory analysis revealed normal liver function tests and serum CEA level but an elevated CA 19-9 level. At operative exploration, the entire transverse colon was found to be retroperitoneal and lies dorsal to the SMA and SMV. The duodenum coursed anterior rather than posterior to the mesenteric vessels. The SMA lies further to the right than normal and the SMV–portal vein confluence lies further to the left than usual. Inspection of the duodenum and head of the pancreas revealed a firm mass in the head of the pancreas. A classic pancreaticoduodenectomy was performed. The anterior placement of the duodenum made division of the jejunum beyond the ligament of Treitz relatively easy. The tumor dissected nicely away from the SMA with no uncinate process of the pancreas present. Our standard procedure is to bring the jejunal limb for reconstruction up in a retrocolic position, through a defect made in the transverse mesocolon to the right of the middle colic vessels. This limb is used to create the pancreaticojejunostomy, hepaticojejunostomy, and gastrojejunostomy. Due to the retroperitoneal position of the transverse colon, we brought the jejunal limb up in an antecolic position. Pathology revealed a moderately differentiated PDA with all surgical margins being free of tumor and 1 out of 11 specimen lymph nodes was positive for metastatic disease. The patient tolerated the procedure well and was discharged to home on postoperative day 5. The patient eventually developed recurrent disease and died 34 months postresection.

## Literature Review

Table 1 shows the eight published cases of HPB malignancy in the setting of abnormalities of gastrointestinal malrotation that have been reported in the English literature from 1957 to the present including the three from this series.^1,4–7^ There were five pancreatic tumors (four ductal adenocarcinomas and one neuroendocrine tumor), two cholangiocarcinomas, and one ampullary adenocarcinoma. Four out of the eight cases involved classic gastrointestinal malrotation where the entire small bowel lies to the right of the superior mesenteric vessels and the colon to the left. In one case, the patient had both classic gastrointestinal malrotation and situs ambiguous and so the entire colon was to the right of the superior mesenteric vessels and the entire small bowel was to the left. Two of the cases had a reversed rotation where the colon was a retroperitoneal structure and lies posterior to the superior mesenteric vessels. Finally, one case had an incomplete rotation where the ascending colon was midline and the duodenojejunal junction was to the right of the midline with some of the distal small bowel present in the left abdomen.

**Table 1.** **Cases of Gastrointestinal Malrotation with Associated HPB Malignancy**

First author	Year	Age	Sex	Congenital abnormality	Associated HPB malignancy	Stage	Presenting symptoms	Management
Daniels	1957	NA	NA	Classic midgut malrotation	PDA	NA	Obstructive jaundice	Pancreaticoduodenectomy
Campbell	1993	60	M	Classic midgut malrotation	PDA	NA	Abdominal pain	Choledochojejunostomy, cholecystectomy, lysis of Ladd's bands, appendectomy
Jagannath	1995	59	M	Reversed rotation	Pancreatic neuroendocrine tumor	NA	Vomiting, constipation	Pancreaticoduodenectomy
Chirica	2005	57	F	Malrotation/situs ambiguous	Intrahepatic cholangiocarcinoma	T1N0Mx	Pruritis, epigastric pain	Hepatic resection, bile duct resection
Mateo	2005	43	M	Classic midgut malroration	Ampullary adenocarcinoma	T2N0M0	Anorexia, obstructive jaundice	Pancreaticoduodenectomy
Case 1	2012	63	M	Classic midgut malrotation	Cholangiocarcinoma	T1N0M0	Nausea, obstructive jaundice	Pancreaticoduodenectomy
Case 2	2012	47	M	Incomplete rotation	PDA	T3N1bM0	Epigastric pain, vomiting	Palliative Roux-en-Y bypass with gastrojejunostomy and hepaticojejunostomy, pancreaticoduodenectomy (second operation)
Case 3	2012	67	F	Reversed Rotation	PDA	T3N1aM0	Epigastric pain, weight loss	Pancreaticoduodenectomy

HPB, hepatopancreatobiliary; PDA, pancreatic ductal adenocarcinoma.

## Discussion

Gastrointestinal malrotation occurs as a failure in the process of normal gastrointestinal rotation and fixation during intrauterine development. Although typically discovered early in a patient's life, malrotation can sometimes quietly persist into adulthood. If symptomatic, adult patients may present with vague symptoms of chronic abdominal pain or with episodes of partial bowel obstruction. At times, adult patients may even present acutely with intestinal volvulus. Although rare, this condition requires clinicians and surgeons to be aware of the unique underlying embryology and anatomic considerations to treat these patients effectively. As our report of these three cases of gastrointestinal malrotation found in the setting of HPB tumors demonstrates, special considerations are required when treating these complex patients.

Mall at Johns Hopkins first described the complex embryogenesis of gastrointestinal development by using embryo models at different stages of development in 1898.^8^ Frazer and Robbins then provided an extensive review of the subject, dividing development into three stages in 1915.^9^ Dott published a review of three cases of various anomalies of intestinal rotation and reviewed the most common rotational anomalies that can arise during each stage of fetal development.^10^

**FIG. 4.**
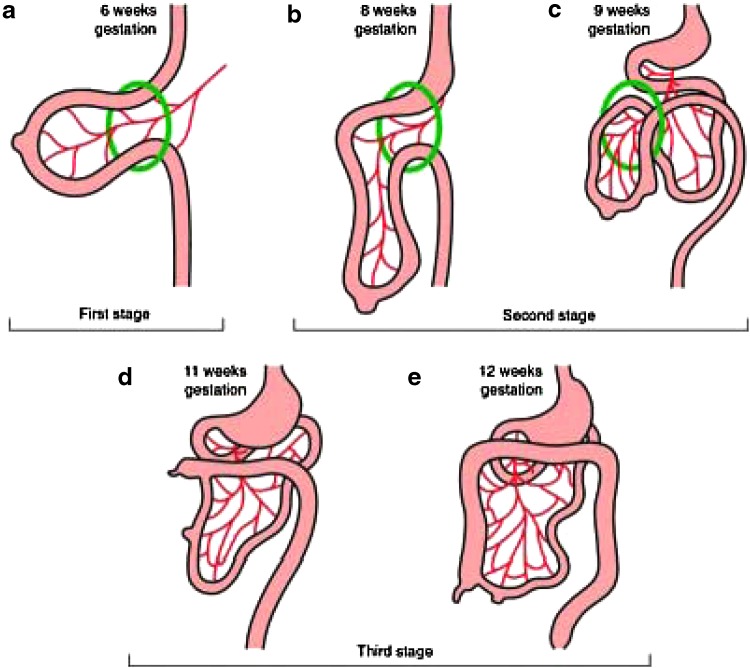
The three stages of gastrointestinal development. Stage 1 **(a)**, extrusion of the gastrointestinal tract; stage 2 **(b, c)**, beginning of a counterclockwise rotation of the gastrointestinal tract and reentry into the abdomen; stage 3 **(d, e)**, finalization of a total of 270° of counterclockwise rotation of the gastrointestinal tract and final elongation of the colon causing descent and fixation of the cecum.

The first stage of gastrointestinal development starts at approximately the fourth week of gestation (Fig. 4a).^11^ At this time, rapid growth of the midgut loop and liver leads to a loss of abdominal domain with a consequent extrusion of the midgut loop into the root of the umbilical cord as a temporary and physiological hernia. The SMA runs from the dorsal aorta between the two segments of the midgut loop to the apex. The vessel divides the midgut loop into a prearterial and postarterial segment with their corresponding mesenteries. At this stage, the loop lies in the sagittal plane with the prearterial segment being anterior and postarterial segment being posterior.^12^ The cecoappendiceal bud appears on the postarterial segment during the fifth week. Whereas in the umbilical cord, the prearterial segment grows faster and longer than the postarterial segment, thereby acquiring a disproportionately longer mesentery. Meanwhile the cecum, ascending and transverse colon develop from the postarterial segment. Derangement of the first stage of gastrointestinal development mainly leads to failure of the viscera to return to the abdominal cavity, which leads to an omphalocele.^2^

The second stage of gastrointestinal rotation occurs at the beginning of the 10th week of development and consists of the rotation and reentry of the gastrointestinal tract back into the abdomen (Fig. 4b, c).^9,11^ The midgut loop returns to the abdominal cavity through the umbilical orifice. The prearterial segment enters first. The small intestine enters the abdomen on the right side of the superior mesenteric vessels and passes to the left side of the abdomen behind the mesenteric vessels.^12^ This movement of the small bowel pushes the hindgut and its mesentery to the left and superior. This will become the transverse and descending colon. After the cecum reenters the abdominal cavity, further elongation of the colon forces it down to the right lower quadrant. The second stage sees a 270° counterclockwise turn around the SMA, which causes the duodenum to cross behind the superior mesenteric vessels near their origin and the colon to cross the same point anteriorly.^10^

One major derangement of the second stage of gastrointestinal development can be failure of rotation of the midgut after returning to the abdominal cavity. This results in the small intestine lying to the right of the midline with the duodenum descending from its normally fixed position to the right of the SMA.^2^ The jejunum and ileum consequently occupy the right lower quadrant. The ileum then enters the cecum in either the midline or the left lower quadrant that brings the duodenum and cecum to rest anterior and superior to the SMA, which leads to a very narrow mesenteric base as in Cases 1 and 2. This is classically characterized as true gastrointestinal malrotation and predisposes to the development of a midgut volvulus. Finally, reversed rotation results from a 90° clockwise rotation that brings the colon in a position dorsal to the SMA as in Case 3.^10^

The third stage of intestinal rotation is characterized by the final elongation of the colon causing descent and fixation of the cecum (Fig. 4d, e).^11^ Both the cecum and duodenum come to rest by fusion of their mesenteries to the posterior parietal peritoneum. Abnormalities of the third stage of intestinal rotation can lead to an excessively floppy cecum, which can predispose patients to the development of a cecal bascule or cecal volvulus.

Previous reports have stated that abnormal visceral rotation occurs in less than 0.2% of patients undergoing exploration for bowel obstruction.^13^ The incidence of abnormal visceral rotation in the setting of HPB malignancy has not yet been reported. We found the prevalence of abnormalities of gastrointestinal rotation to be 0.2% in the setting of HPB tumors at our own institution.

**FIG. 5.**
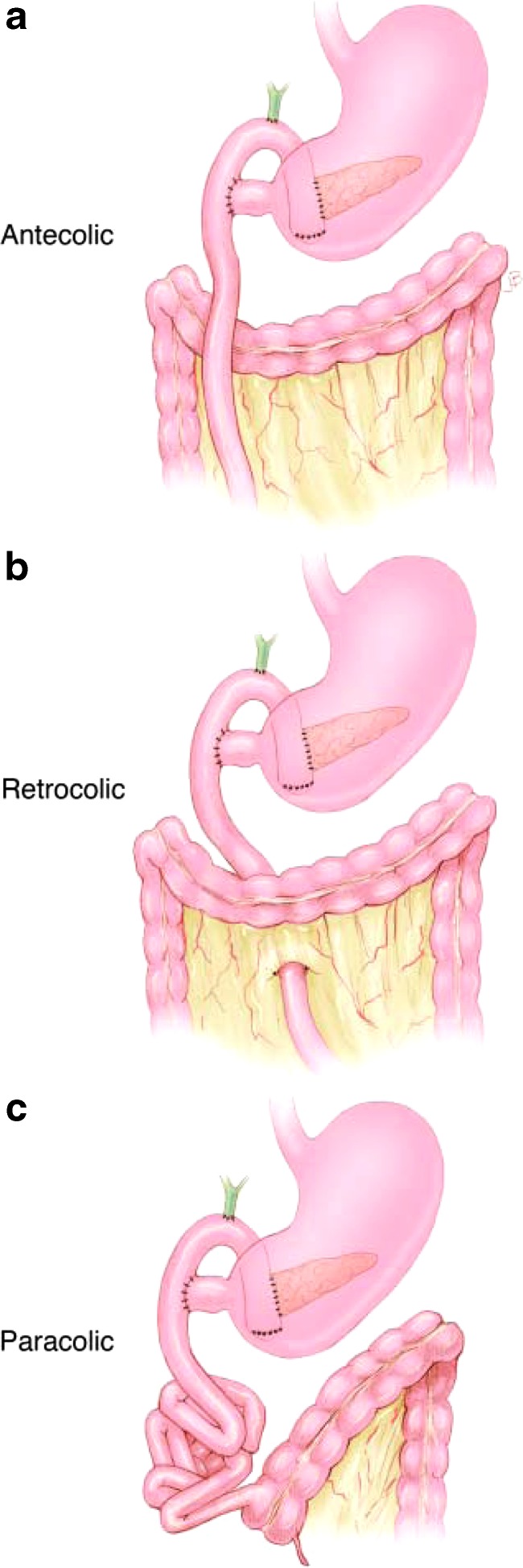
Illustrations showing different reconstructive positions of the Roux limb in relation to the colon. **(a)** Antecolic position of the jejunal limb. **(b)** Retrocolic position of the jejunal limb. **(c)** Paracolic position of the jejunal limb.

Patients who have abnormal gastrointestinal rotation with an HPB cancer can present in a manner similar to patients with HPB malignancy alone. Vague abdominal discomfort accompanied by symptoms of biliary and visceral obstruction typically existed for more than several months in our patients. The abnormal visceral anatomy was identified preoperatively in all three of our patients. Abnormal abdominal visceral anatomy has an impact on the operation both in the challenges that it poses in identifying abdominal vasculature during oncological resection and in reconstruction after resection. Case 3 had a reversed rotation with a retroperitoneal colon and the duodenum anterior to the superior mesenteric vessels. We usually bring the jejunal limb up in a retrocolic position (Fig. 5b). The retroperitoneal position of the colon prevented this and required us to bring the jejunal limb up in an antecolic position (Fig. 5a). The other two patients had the most commonly found anatomy of gastrointestinal malrotation, with the small bowel entirely on the right and the colon on the left of the superior mesenteric vessels. Cases 1 and 2 required the jejunal limb to be brought up for the pancreaticojejunostomy and hepaticojejunostomy in a paracolic manner (Fig. 5c). The limb was also tacked to the retroperitoneum to protect against internal hernia formation.

We and others have shown that resection for HPB malignancy in the setting of abnormal visceral rotation can be performed safely. In all of the cases, the abnormal intestinal rotation was discovered on preoperative imaging. There are hallmark features on CT or MRI that the HPB surgeon should look for, so as to be prepared for these rotational abnormalities. A classic malrotation exists when the small intestine is in a position to the right of the midline and the duodenum descending from its normally fixed position to the right of the SMA. A reversed rotation exists when the colon is in a position dorsal to the SMA. Great care and consideration must go into operative planning and technique for a safe dissection and reconstruction when these abnormalities of gastrointestinal rotation are found on preoperative imaging.

In summary, our case series demonstrates that with an adequate understanding of typical anatomic abnormalities of gastrointestinal malrotation, procedures for HPB malignancies can be safely performed with appropriate adjustments in the operative technique.

## Abbreviations Used

CA: cancer antigen
CEA: carcinoembryonic antigen
CT: computed tomography
HPB: hepatopancreatobiliary
MRI: magnetic resonance imaging
PDA: pancreatic ductal adenocarcinoma
SMA: superior mesenteric artery
SMV: superior mesenteric vein
